# miRNA-34c-5p inhibits amphiregulin-induced ovarian cancer stemness and drug resistance via downregulation of the AREG-EGFR-ERK pathway

**DOI:** 10.1038/oncsis.2017.25

**Published:** 2017-05-01

**Authors:** S-L Tung, W-C Huang, F-C Hsu, Z-P Yang, T-H Jang, J-W Chang, C-M Chuang, C-R Lai, L-H Wang

**Affiliations:** 1 Institute of Molecular Medicine, National Tsing Hua University, Hsinchu, Taiwan; 2 Institute of Molecular and Genomic Medicine, National Health Research Institutes, Miaoli, Taiwan; 3 Department of Hematology and Oncology, Ton-Yen General Hospital, Hsinchu, Taiwan; 4 Institute of Clinical Medicine, School of Medicine, National Yang-Ming University, Taipei, Taiwan; 5 Division of Genetics and Prenatal Diagnosis, Department of Obstetrics and Gynecology, Taipei Veterans General Hospital, Taipei, Taiwan; 6 Department of Medicine, School of Medicine, National Yang-Ming University, Taipei, Taiwan; 7 Section of Cytopathology, Department of Pathology, Taipei Veterans General Hospital, Taipei, Taiwan; 8 China Medical University, Taichung, Taiwan

## Abstract

Epithelial ovarian cancer is the most lethal gynecological cancer mainly due to late diagnosis, easy spreading and rapid development of chemoresistance. Cancer stem cells are considered to be one of the main mechanisms for chemoresistance, as well as metastasis and recurrent disease. To explore the stemness characteristics of ovarian cancer stem cells, we successfully enriched ovarian cancer stem-like cells from an established ovarian cancer cell line (SKOV-I6) and a fresh ovarian tumor-derived cell line (OVS1). These ovarian cancer stem-like cells possess important cancer stemness characteristics including sphere-forming and self-renewing abilities, expressing important ovarian cancer stem cell and epithelial–mesenchymal transition markers, as well as increased drug resistance and potent tumorigenicity. Microarray analysis of OVS1-derived sphere cells revealed increased expression of amphiregulin (AREG) and decreased expression of its conserved regulatory microRNA, miR-34c-5p, when compared with the OVS1 parental cells. Overexpression of AREG and decreased miR-34c-5p expression in SKOV-I6 and OVS1 sphere cells were confirmed by quantitative real-time PCR analysis. Luciferase reporter assay and mutant analysis confirmed that AREG is a direct target of miR-34c-5p. Furthermore, AREG-mediated increase of sphere formation, drug resistance toward docetaxel and carboplatin, as well as tumorigenicity of SKOV-I6 and OVS1 cells could be abrogated by miR-34c-5p. We further demonstrated that miR-34c-5p inhibited ovarian cancer stemness through downregulation of the AREG-EGFR-ERK pathway. Overexpression of AREG was found to be correlated with advanced ovarian cancer stages and poor prognosis. Taken together, our data suggest that AREG promotes ovarian cancer stemness and drug resistance via the AREG-EGFR-ERK pathway and this is inhibited by miR-34c-5p. Targeting AREG, miR-34c-5p could be a potential strategy for anti-cancer-stem cell therapy in ovarian cancer.

## Introduction

Epithelial ovarian cancer is the most lethal gynecological cancer.^1, 2^ Its high mortality rate is mainly due to late diagnosis, easy spreading, and rapid development of chemoresistance.^1, 2, 3^ Cancer stem cells are considered to be in part account for chemoresistance, as well as metastasis and recurrent disease.^4, 5, 6^ Cancer stem cells are defined as a very small subpopulation of tumor cells possessing the ability to self-renew and differentiate leading to the formation of heterogeneous progeny forming the tumor.^4, 7^ Although number of unique genes and microRNAs (miRNAs) have been found to regulate ovarian cancer stem cells, effective and clinically applicable inhibitors against ovarian cancer stem cells are yet to be developed.^8, 9, 10, 11, 12, 13, 14^

Human amphiregulin (AREG) is a glycoprotein composed of 84 amino acids and is one of the ligands for the epidermal growth factor receptor (EGFR), a widely expressed transmembrane protein tyrosine kinase.^15, 16^ Via binding to EGFR and triggering EGFR signaling, AREG has been reported to have important roles in oncogenesis including inhibition of apoptosis, promotion of proliferation, migration, invasion, angiogenesis, chemoresistance and metastasis through activating various downstream signaling pathways such as MAPK/ERK, PI3K/AKT, mTOR and STAT pathways.^15, 16^ Overexpression of AREG has been reported in solid tumors including ovarian cancer.^15, 16^ However, the role of AREG in cancer stemness has never been reported. Information about regulation of AREG expression by miRNAs in solid tumors is also scarce.^17, 18, 19, 20^

The role of miRNAs, a conserved class of small non-coding RNAs consisting of 21-25 nucleotides in length, in anti-cancer therapy has been actively pursued in recent years.^21, 22, 23, 24^ By binding to the mRNA 3′-untranslated region (3′UTR) sequences of their target genes, miRNAs have been reported to modulate numerous oncogenes or tumor suppressor genes as well as to positively or negatively regulate cancer stem cells.^22, 23, 24, 25^ Although several laboratories have explored the suppressor roles of miR-34 family in cancer stem cells of various solid tumors such as colon, breast, pancreas, prostate, glioma and non-small cell lung cancer,^24, 26, 27, 28, 29^ the role of miR-34 family in ovarian cancer stem cells is still unknown.

In present study, we successfully enriched ovarian cancer stem-like cells from an established human ovarian cancer cell line (SKOV-I6) and a fresh ovarian tumor-derived cell line (OVS1). Altered expression levels of AREG and miR-34c-5p were found in those ovarian cancer stem-like cells. The effects of AREG and miR-34c-5p on ovarian cancer stemness and drug resistance were investigated for the first time. Our study has demonstrated that AREG has an important role in promoting ovarian cancer stemness and drug resistance. We are also the first to identify that miR-34c-5p inhibits ovarian cancer stemness and drug resistance through downregulation of the AREG-EGFR-ERK pathway. These results provided important evidence to support miR-34c-5p and AREG as promising candidates for anti-cancer-stem cell therapy in ovarian cancer.

## Results

### Successful enrichment of ovarian cancer stem-like cells from both SKOV-I6 and OVS1 lines

**Figure 1:**
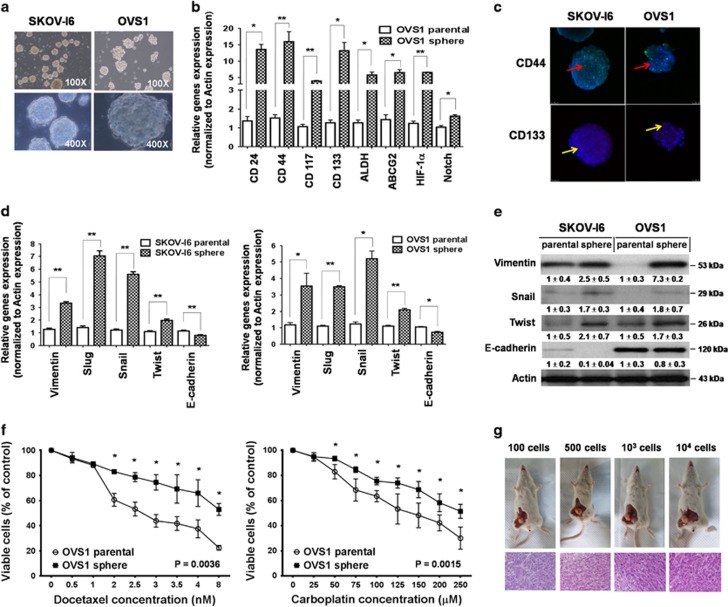
Ovarian cancer stem-like cells were successfully enriched from SKOV-I6 and OVS1 cell lines. (**a**) Formation of spheres under the stem cell selective condition on day 8 after culturing from parental SKOV-I6 and OVS1 cells is shown. (**b**) The mRNA expression levels of ovarian cancer stem cell markers in parental OVS1 cells and their sphere cells were analyzed by qRT–PCR with actin as an internal control. Histograms represent means±s.d. from three independent experiments (*P<0.05, **P<0.01). (**c**) SKOV-I6 and OVS1 sphere cells expressed ovarian cancer stem cell markers CD44 and CD133 as shown in confocal immunofluorescence images. CD44 (red arrows) and CD133 (yellow arrows) were detected on their surfaces. The nuclei were stained with DAPI. (**d**) The mRNA expression levels of EMT markers in parental SKOV-I6 and OVS1 cells and their sphere cells were analyzed by qRT–PCR with actin as an internal control. Histograms represent means±s.d. from three independent experiments (*P<0.05, **P<0.01). (**e**) The protein expression levels of EMT markers in parental SKOV-I6 and OVS1 cells and their sphere cells were analyzed by western blotting with β-actin as an internal control. Relative band intensity was quantified by ImageJ 1.42 (Windows version of NIH Image, http://rsb.info.nih.gov/ij/) and was represented with normalized mean±s.e. (*N*=3) below each band. (**f**) Dose-dependent growth inhibition of parental OVS1 cells and their sphere cells upon continuous exposure to the indicated concentrations of docetaxel or carboplatin for 48 h was measured by MTT assay. Each dosage point represent the mean±s.e. from three independent experiments (*P<0.05). (**g**) Top, representative xenograft tumors formed by different numbers of OVS1 sphere cells in the NOD/SCID mice. Bottom, the histopathology of these xenograft tumors by H&E staining and were characterized as adenocarcinoma.

In order to enrich for cancer stem-like cells, parental SKOV-I6 and OVS1 cells from monolayer were cultured in a stem cell selective condition described in ‘Materials and Methods’ to form spheres. Some of the suspended cells underwent apoptosis during the first 2 days of culturing, and the rest of survived cells formed floating spheres gradually. The spheres grew larger and assume confluent rounded three-dimensional configuration and often reached to 50–100 μM in diameter after 5–8 days (Figure 1a). Spheres were then harvested and propagated. The cells dispersed from those spheres could form new spheres in the subsequent culture. Both SKOV-I6 and OVS1 spheres could be serially passaged for more than 4–5 generations with effective sphere-forming ability, which indicated their unique ability to self-renew.^30, 31^

Both SKOV-I6 and OVS1 sphere-dispersed cells (sphere cells) had elevated expression of ovarian cancer stem cell and epithelial–mesenchymal transition (EMT) markers mRNAs compared with their parental cells. These ovarian cancer stem cell markers, including CD24, CD44, CD117, CD133 and ALDH, as well as multidrug transporters ABCG2, were significantly higher in SKOV-I6 and OVS1 sphere cells shown by quantitative real-time polymerase chain reaction (qRT–PCR) analysis (Figure 1b; Supplementary Figure 1).^32, 33, 34, 35^ The mRNA levels of cancer stem cell maintenance factors such as HIF1-α and Notch were also significantly higher in SKOV-I6 and OVS1 sphere cells than in their parental cells (Figure 1b; Supplementary Figure 1).^36, 37, 38^ We also performed immunofluorescence staining to assess the level of CD44 and CD133 in SKOV-I6 and OVS1 spheres. Diverse expression pattern of CD44 and CD133 was found in spheres of both cell lines (Figure 1c). As for the EMT markers, overexpression of mesenchymal markers including Vimentin, Slug, Snail and Twist as well as loss of expression of the epithelial marker, E-cadherin,^39, 40^ was found in SKOV-I6 and OVS1 sphere cells at the transcriptional and translational levels (Figures 1d and e).

We examined the chemosensitivity of theses sphere cells and their parental cells by using two first-line chemotherapeutic agents for treating ovarian cancer, docetaxel and carboplatin.^41^ Parental SKOV-I6 and OVS1 cells and their sphere cells were treated with docetaxel and carboplatin separately for 48 h and cell viability was measured by MTT assay. Sphere cells from both cell lines were more chemoresistant than their respective parental cells. Higher percentage of viable cells of SKOV-I6 sphere cells at the IC_50_ level of docetaxel (8 nM) and carboplatin (100 μM) indicated significantly increased chemoresistance (Supplementary Figure 2). Similar results were obtained with OVS1 sphere cells at the IC_50_ level of docetaxel (2.5 nM) and carboplatin (150 μM) (Supplementary Figure 2). SKOV-I6 and OVS1 sphere cells are more chemoresistant to continuous exposure to various concentrations of docetaxel and carboplatin (Figure 1f; Supplementary Figure 3). Thus SKOV-I6 and OVS1 sphere cells showed significantly increased chemoresistance toward docetaxel and carboplatin.

To investigate the tumorigenic potential of OVS1 sphere cells, four different cell numbers (100, 500, 10^3^, 10^4^ cells) of parental OVS1 cells or their sphere cells were implanted into five NOD.CB17-*Prkdc*^scid^/NcrCrl (NOD/SCID) mice for each group. Tumor growth was noticed in mice implanted with OVS1 sphere cells even as few as 100 cells with tumor latency of 135 days (Table 1). None of the mice implanted with OVS1 parental cells formed xenograft tumors (Table 1). All xenograft tumors derived from OVS1 sphere cells were resected for pathological examination and were categorized as adenocarcinoma resembling the tumor phenotype of the fresh ovarian tumor which OVS1 cell line was derived from (Figure 1g; Supplementary Figure 4).

Taken together, we have successfully enriched ovarian cancer stem-like cells from both SKOV-I6 and OVS1 cell lines and they display hallmark cancer stem cell characteristics including sphere-forming and self-renewing ability, expression of ovarian cancer stem cell and EMT markers, more chemoresistant and potent tumorigenicity in accordance with established parameters of cancer stem-like cells.^4, 7, 30, 31, 42^

### Upregulated AREG and downregulated miR-34c-5p expression were observed in SKOV-I6 and OVS1 sphere cells

**Figure 2:**
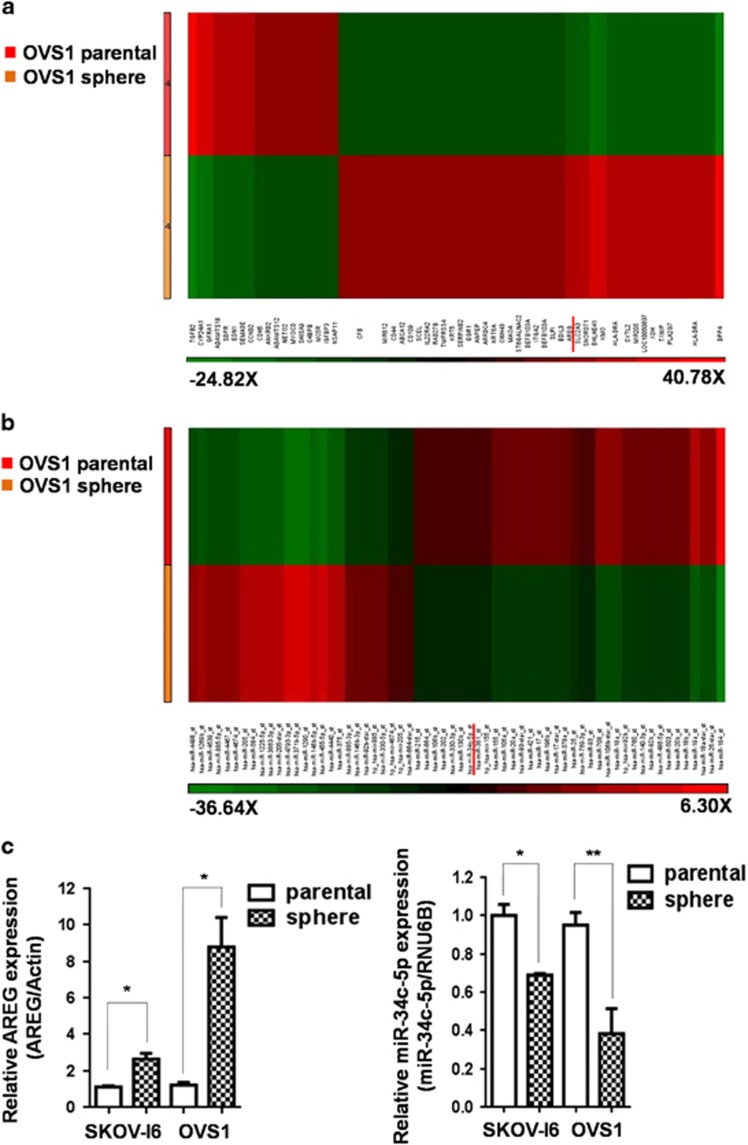
Upregulated AREG and downregulated miR-34c-5p expression levels were observed in SKOV-I6 and OVS1 sphere cells. (**a**) Genome-wide gene expression in OVS1 cell line was analyzed by microarray analysis. The expression of AREG is 6.94-fold upregulated in OVS1 sphere cells compared with their parental cells. The ranges of fold are listed below the figure. (**b**) Genom-wide microRNAs expression in OVS1 cell line was analyzed by microarray analysis. The expression of miR-34c-5p in OVS1 sphere cells showed -2.26-fold change compared with their parental cells. The ranges of fold are listed below the figure. (**c**) The mRNA expression levels of AREG and miR-34c-5p in parental SKOV-I6 and OVS1 cells and their sphere cells were analyzed by qRT–PCR. Actin and RNU6B were used as internal controls respectively. Histograms represent means±s.d. from three independent experiments (*P<0.05; **P<0.01).

To elucidate the expression of potential genes and miRNAs related to ovarian cancer stemness, genome-wide gene and miRNA microarray analyses of parental OVS1 cells and their sphere cells were performed for transciptome profiling (Figures 2a and b). Among upregulated genes with threshold of 5-fold change of expression level in OVS1 sphere cells compared with their parental cells, we performed literature searching and found 11 cancer stemness related genes and their conserved miRNAs predicted by TargetScan 7.0 (Supplementary Table 1). Among these predicted miRNAs, four were found in our miRNA microarray data (Supplementary Table 2). Among the four, three were upregulated and miR-34c-5p in OVS1 sphere cells showed downregulated expression to -2.26-fold change compared with their parental cells, namely the level of miR-34c-5p in OVS1 sphere cells was about 44% of that of their parental cells. (Figure 2b; Supplementary Table 2). Other miR-34 family members did not display downregulation in OVS1 sphere cells. MiR-34a showed 2.51-fold upregulation (Supplementary Table 2) and miR-34b showed no significant change in OVS1 sphere cells (data not shown). We performed qRT–PCR analysis and found no significant increase of miR-34a mRNA level in OVS1 sphere cells, although there appeared to have a trend of increase in SKOV-I6 sphere cells yet without statistical significance (Supplementary Figure 5a). Furthermore, qRT–PCR analysis showed that transfection of miR-34a failed to cause a significant reduction of AREG in SKOV-I6 or OVS1 cells (Supplementary Figure 5b). Because the expression level of miR-34a was inconsistent in qRT–PCR analysis and in microarray data in OVS1 sphere cells, and miR-34a also failed to show inhibitory effects on AREG in both cell lines, we did not further explore other functional roles of miR-34a. On the other hand, AREG, the target gene of miR-34c-5p, was found with increased expression of 6.94-fold in OVS1 sphere cells compared with their parental cells (Figure 2a).

We further confirmed the upregulation of AREG at transcriptional level by qRT–PCR in SKOV-I6 and OVS1 sphere cells compared with their parental cells (Figure 2c). Transcriptional downregulation of miR-34c-5p was also confirmed by qRT–PCR in SKOV-I6 and OVS1 sphere cells (Figure 2c).

### AREG is a direct target of miR-34c-5p

**Figure 3:**
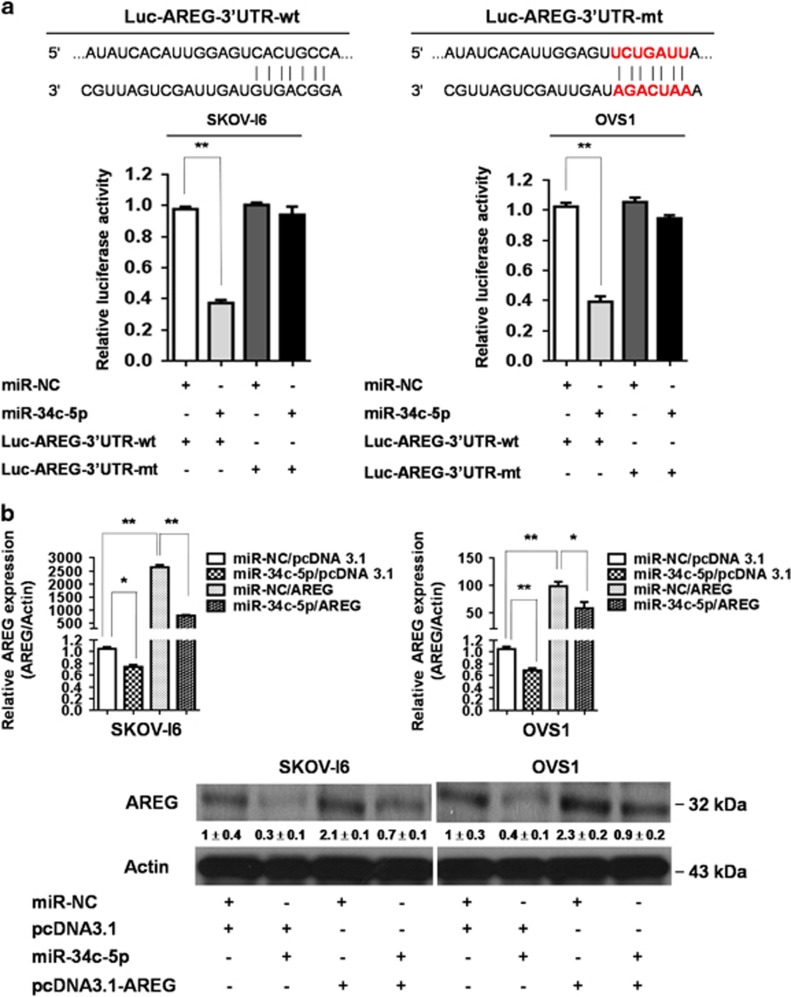
Identification of AREG as the direct target of miR-34c-5p. (**a**) Effect of miR-34c-5p on Luc-AREG-3′UTR-wt (wild type) and Luc-AREG-3′UTR-mt (mutant) luciferase reporters in SKOV-I6 and OVS1 cells. Top, the Luc-AREG-3′UTR-wt sequence and the Luc-AREG-3′UTR-mt sequence in which the sequence in red was mutagenized to abolish the binding between miR-34c-5p and AREG-3′UTR. Bottom, Luciferase reporter assay showed decreased activity of more than 50% after cotransfection with miR-34c-5p and Luc-AREG-3′UTR-wt in SKOV-I6 and OVS1 cells. The activity of Luc-AREG-3′UTR-mt was not significantly affected by miR-34c-5p. Histograms represent means±s.d. from three independent experiments (*P<0.05, **P<0.01). (**b**) miR-34c-5p inhibited AREG in both mRNA and protein expression levels in SKOV-I6 and OVS1 cells. Top, the mRNA expression levels of AREG in both cell lines were measured by qRT–PCR from cells transfected with the indicated plasmids. Histograms represent means±s.d. from three independent experiments (*P<0.05, **P<0.01). Bottom, the protein expression levels as reflected by western blotting of AREG in both cell lines transfected with the indicated plasmids are shown. β-actin was used as an internal control. Relative band intensity was quantified by ImageJ 1.42 and was represented with normalized mean±s.e. (*N*=3) below each band.

To validate whether AREG is a direct target of miR-34c-5p, we constructed the wild-type Luciferase-AREG-3′UTR (Luc-AREG-3′UTR-wt) plasmid and its mutant plasmid (Luc-AREG-3′UTR-mt) which the putative miR-34c-5p binding site was mutated (Figure 3a). Luciferase reporter assay showed that luciferase activity of the Luc-AREG-3′UTR-wt reporter was suppressed more than 50% by miR-34c-5p in SKOV-I6 and OVS1 cells compared with the control. In contrast, miR-34c-5p had little suppression effect on the luciferase activity of the Luc-AREG-3′UTR-mt reporter (Figure 3a).

Moreover, qRT–PCR and western blotting analysis confirmed that transfection of miR-34c-5p caused a significant reduction of AREG in both transcriptional and translational levels either in SKOV-I6 or in OVS1 cells (Figure 3b). These results indicate that miR-34c-5p directly targets AREG resulting in downregulation of its mRNA and protein.

### AREG promotes SKOV-I6 and OVS1 sphere formation, tumorigenicity and drug resistance, which are inhibited by miR-34c-5p

AREG has been shown to have important roles in promoting cancer development and increasing drug resistance.^16, 43, 44, 45^ AREG was also reported to promote mammospheres formation and mediates self-renewal in an immortal mammary cell line with stem cell characteristics.^46^ Therefore we decided to further explore the functional roles of AREG and miR-34c-5p in ovarian cancer stemness and drug resistance.

**Figure 4:**
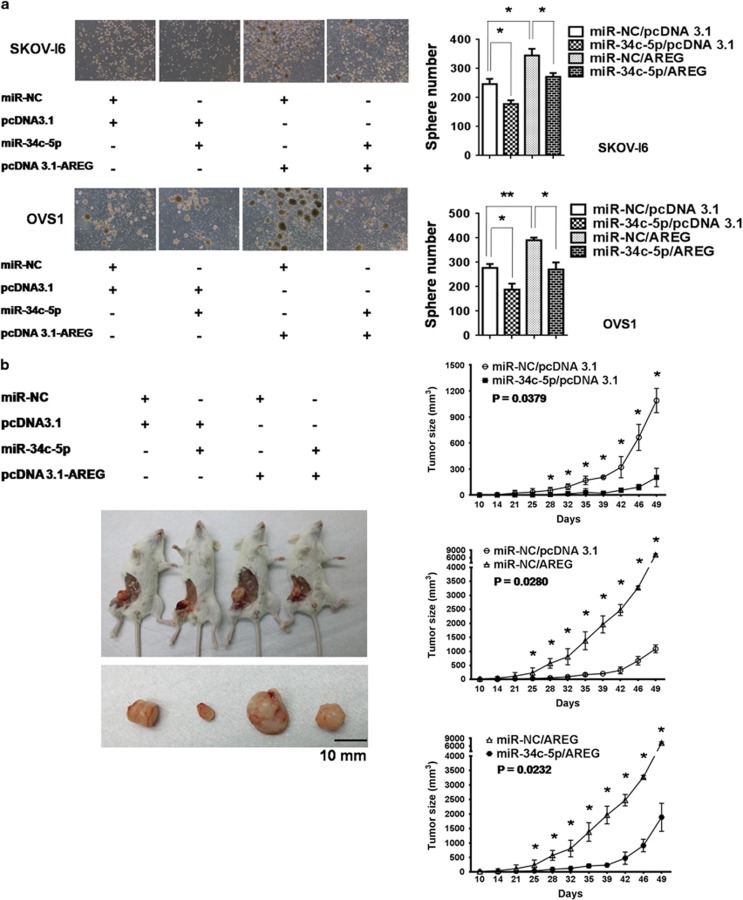
The effects of AREG and miR-34c-5p on sphere formation and tumorigenicity. (**a**) AREG-augmented sphere-forming ability was suppressed by miR-34c-5p in SKOV-I6 and OVS1 cells. Left, sphere formation under the stem cell selective condition was observed on day 10 after culturing from cells transfected with the indicated plasmids. Right, sphere numbers were counted on day 10 after culturing. Histograms represent means±s.d. from three independent experiments (*P<0.05, **P<0.01). (**b**) AREG-augmented tumorigenicity was inhibited by miR-34c-5p. Left, SKOV-I6 parental cells were categorized into four groups according to the transfection of plasmids as indicated. 1 × 10^6^ cells in each group were implanted into five NOD/SCID mice separately. Tumor growth was monitored. One mouse carrying the tumor with representative size of each group was sacrificed at day 32 after implantation. Right, comparison of tumor growth. All mice were sacrificed on day 49 after implantation. Each time point represents the mean±s.e. of three xenograft tumors in each group (*P<0.05).

First, we tested whether sphere-forming ability of SKOV-I6 and OVS1 cells could be promoted by AREG and suppressed by miR-34c-5p. After 10 days culturing of SKOV-I6 and OVS1 cells in the stem cell selective condition, sphere number was calculated by visual counting under microscope. Overexpression of AREG caused significant increase of sphere number whereas transfection of miR-34c-5p reduced it markedly, cotransfection with AREG and miR-34c-5p nullified the promoting effect of AREG (Figure 4a). Thus, our data strongly suggest that miR-34c-5p targets AREG to inhibit its promotion of sphere formation.

Next, we examined the enhancing and inhibitory effect of AREG and miR-34c-5p, respectively, on tumorigenicity. Monitoring of tumor growth showed that AREG markedly increased tumorigenicity, which was suppressed by miR-34c-5p significantly (Figure 4b). Cotransfection with AREG and miR-34c-5p reverted the tumor promoting effect of AREG (Figure 4b).

**Figure 5:**
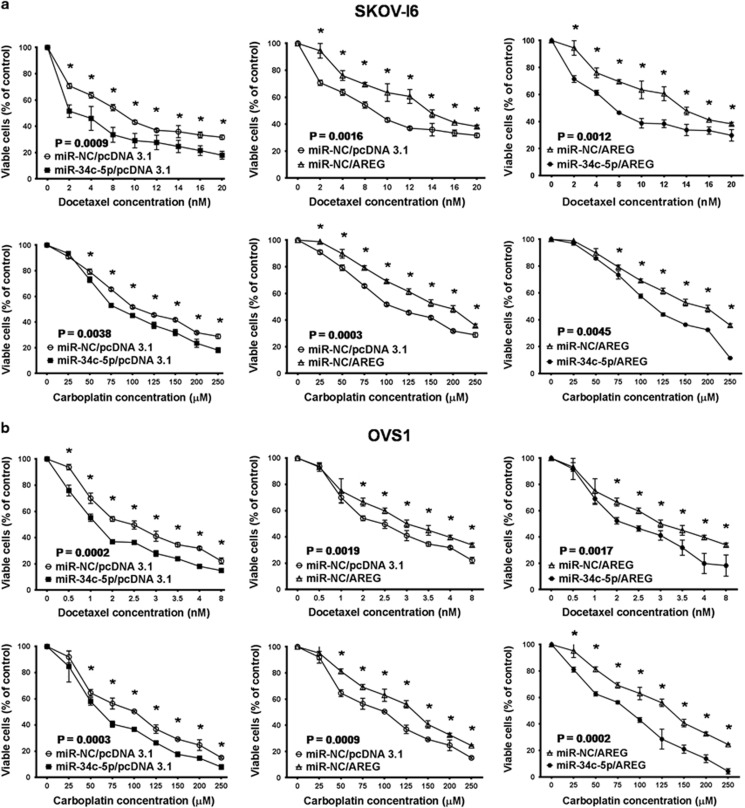
The effects of AREG and miR-34c-5p on drug resistance. (**a**) Dose-dependent growth inhibition of parental SKOV-I6 cells upon continuous exposure to the indicated concentrations of docetaxel or carboplatin for 48 h was measured by MTT assay. Cells were divided into four groups by transfection with the indicated plasmids. Each dosage point represents the mean±s.e. from three independent experiments (*P<0.05). (**b**) Similar experiments were carried out with parental OVS1 cells. Each dosage point represents the mean±s.e. from three independent experiments (*P<0.05).

We next investigated the impact of AREG and miR-34c-5p on drug resistance. In parental SKOV-I6 and OVS1 cells, we found that cell viability at docetaxel and carboplatin IC_50_ levels was decreased after transfection with miR-34c-5p and increased upon overexpression of AREG (Supplementary Figure 6). The increase of cell viability at IC_50_ levels by AREG was reduced by cotransfection with miR-34c-5p (Supplementary Figure 6). SKOV-I6 and OVS1 cells became more chemosensitive to continuous exposure to different doses of docetaxel and carboplatin after transfection with miR-34c-5p, whereas overexpression of AREG increased chemoresistance, which was reverted by cotransfection with miR-34c-5p (Figures 5a and b).

### miR-34c-5p inhibits ovarian cancer stemness and drug resistance through downregulation of the AREG-EGFR-ERK pathway

The effect of EGFR-ERK pathway on cell survival, proliferation, motility and drug resistance has been extensively studied.^47, 48^ Considering AREG is the ligand of EGFR and the promotion effects of AREG on ovarian cancer stemness and drug resistance, we further investigated if miR-34c-5p inhibited ovarian cancer stemness and drug resistance through the AREG-EGFR-ERK axis.

**Figure 6:**
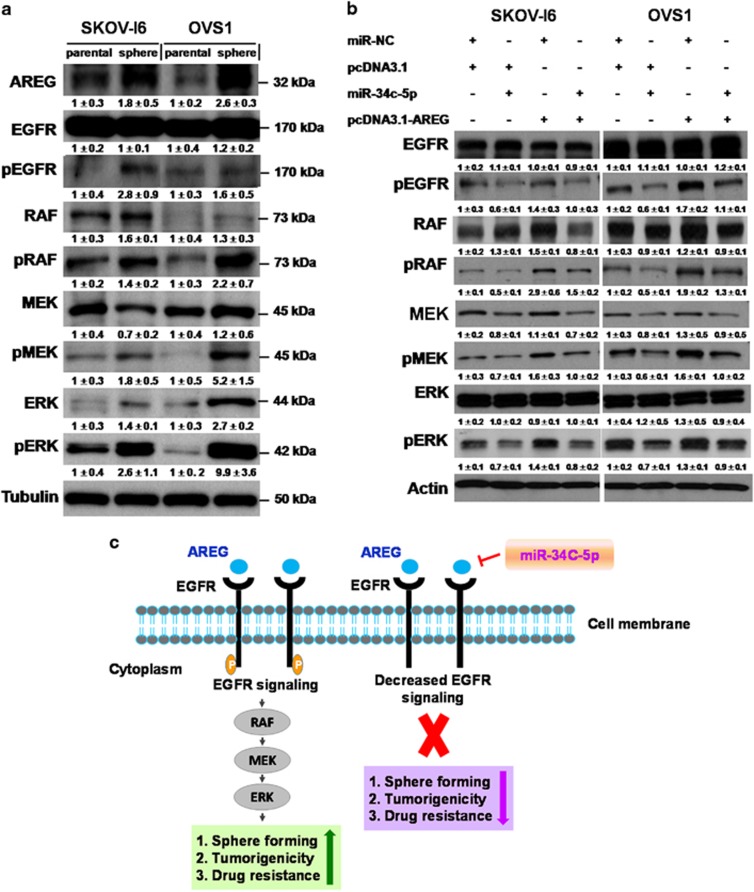
miR-34c-5p inhibits AREG via downregulation of the AREG-EGFR-ERK pathway. (**a**) The protein expression levels of the signaling components of the AREG-EGFR-ERK pathway in parental SKOV-I6 and OVS1 cells and their sphere cells are shown by western blotting. Tubulin was used as an internal control. Relative band intensity was quantified by ImageJ 1.42 and was represented with normalized mean±s.e. (*N*=3) below each band. (**b**) The protein expression levels of the signaling components of the AREG-EGFR-ERK pathway in parental SKOV-3-I6 and OVS1 cells transfected with the indicated plasmids are shown by western blotting. β-actin was used as an internal control. Relative band intensity was quantified by ImageJ 1.42 and was represented with normalized mean±s.e. (*N*=3) below each band. (**c**) The model of signaling pathway that depicts miR-34c-5p targeting AREG to inhibit the AREG-EGFR-ERK signaling and to affect ovarian cancer stemness and drug resistance.

By western blotting analysis, we found increase of several proteins in the AREG-EGFR-ERK pathway including AREG and phosphorylated forms of EGFR, Raf, MEK and ERK in SKOV-I6 and OVS1 sphere cells compared with their parental cells (Figure 6a). Upregulation of AREG protein in SKOV-I6 and OVS1 sphere cells were compatible with upregulated level of AREG mRNA shown by qRT–PCR (Figures 2c and 6a). This confirms that the AREG-EGFR-ERK pathway is upregulated in ovarian cancer stem-like cells and implies that AREG regulates ovarian cancer stemness and drug resistance through the AREG-EGFR-ERK pathway.

We then examined if miR-34c-5p inhibited ovarian cancer stemness and drug resistance through downregulation of the AREG-EGFR-ERK pathway. Via western blotting analysis, we found that the expression of phosphorylated forms of EGFR, Raf, MEK, and ERK proteins increased after transfection with AREG and decreased after transfection with miR-34c-5p in SKOV-I6 and OVS1 cells (Figure 6b). The enhanced expression of phosphorylated proteins of the AREG-EGFR-ERK pathway caused by AREG was reverted by cotransfection with miR-34c-5p (Figure 6b). It is concluded that miR-34c-5p inhibits AREG-augmented ovarian cancer stemness and drug resistance through downregulation of the AREG-EGFR-ERK pathway (Figure 6c).

### The expression of AREG in ovarian cancer patients correlates with advanced clinical stages and poor clinical outcomes

**Figure 7:**
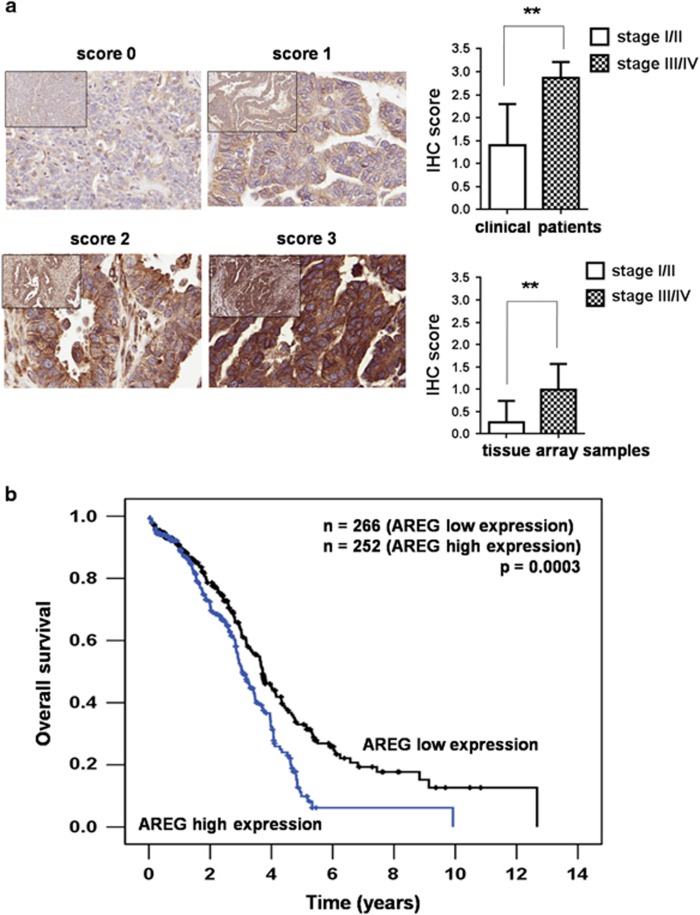
The expression level of AREG in ovarian cancer specimens correlates with clinical staging and overall survival of the patients. (**a**) Left, representative examples of the expression levels of AREG protein determined by IHC (scores 0–3) of clinical specimens and commercial tissue array samples. The 65 paraffin-embedded clinical specimens were obtained from the Department of Pathology, Taipei Veterans General Hospital. Tumor samples were collected during debulking surgery between 2012 and 2015. The commercial tissue array slide (OV6161) was purchased from US Biomax Inc. (Rockville, MD, USA) and contained 280 cases of ovarian adenocarcinoma specimens. Right, the correlation of IHC score of AREG protein expression and ovarian cancer stages from patients and the commercial tissue array samples. Histograms represent means±s.d. from three independent experiments (*P<0.05, **P<0.01). (**b**) The mRNA expression level of AREG correlates with overall survival in 518 ovarian cancer patients as calculated from that data in Oncomine.

We further explored the correlation of AREG expression level and clinical outcomes of ovarian cancer patients. The paraffin-embedded specimens from clinical patients and commercial tissue array samples were stained with AREG antibody by immunohistochemistry (IHC). Higher IHC score of AREG protein expression correlated with the advanced stages of ovarian cancer in both clinical patients and the tissue array samples (Figure 7a). In survival study, Kaplan–Meier survival analysis of 518 cases from the Oncomine data showed that ovarian cancer patients with high AREG expression correlated with a significantly shorter survival than those with low AREG expression (*P*=0.0003, Figure 7b). The results suggest that the expression of AREG in ovarian cancer patients correlates with poor clinical outcomes and could serve as an important prognostic marker.

## Discussion

Rapid development of chemoresistance is a main obstacle in treating epithelia ovarian cancer.^2^ Most ovarian cancer patients in advanced stages will develop recurrence within 18 months despite previous treatment and result in chemoresistance and dismal 5-year survival.^2, 49^ Although platinum-based chemotherapy is the main stream in the treatment of ovarian cancer, many patients eventually became platinum refractory with poor prognosis.^2^ Other therapeutic agents, including pegylated liposomal doxorubicin, topotecan, bevacizumab and olaparib, have been developed but only with modest effect in the recurrent disease.^50, 51, 52, 53^ As cancer stem cells contribute to one of the main mechanisms of chemoresistance and likely also recurrence, identification of targetable molecular markers and miRNAs regulating ovarian cancer stem cells will be important to improve treatment efficacy. In the present study, we successfully enriched ovarian cancer stem-like cells and demonstrated that AREG promotes ovarian cancer stemness and drug resistance via the AREG-EGFR-ERK pathway and that miR-34c-5p targets AREG to inhibit ovarian cancer stemness and drug resistance. Both AREG and miR-34c-5p could potentially serve as prognostic biomarkers and/or targets for developing ovarian cancer therapeutics.

Cancer stem cells are well known to possess several important properties including the ability to grow into three-dimensioned spheres, self-renewal ability, generating xenograft tumors with high potency, and expression of unique cancer stem cell surface markers.^4, 7, 30, 31, 42^ Common methods for enrichment of ovarian cancer stem cells include selection by cancer stem cell surface markers, sphere forming and drug treatment.^32, 54, 55^ Our study enriched ovarian cancer stem-like cells from SKOV-I6 and OVS1 cell lines by sphere-forming method in nonadhesive culture plates with serum-free culture medium. These enriched spheroid cells expressed hallmark characteristics of ovarian cancer stem cells as described above and formed tumor with histology resembling the original tumor phenotype which OVS1 cell line was derived from. These ovarian cancer stem-like cells overexpressed specific ovarian cancer stem cell markers including CD24, CD44, CD117, CD133 and ALDH. Although some studies isolated ovarian cancer stem cells by selecting CD24^−^ cells,^33, 34, 56^ most studies support CD24 as an important ovarian cancer stem cell marker.^33, 34, 35, 57^ We also demonstrated that the ovarian cancer stem-like cells possess EMT markers and significant chemoresistance in accordance with previous reports.^34, 54, 58^ These results confirmed that we had successfully enriched ovarian cancer stem-like cells from both cell lines.

Although AREG was reported to have a pivotal role for mammary gland ductal morphogenesis and to mediate oncogenic processes,^15, 16^ only one study reported that AREG enhanced mammospheres with stem cell characteristics from mammary epithelial cells.^46^ The role of AREG in cancer stem cells has never been clearly demonstrated before. Our investigation for the first time demonstrated that AREG was upregulated in ovarian cancer stem-like cells and contributed to ovarian cancer stemness characteristics. Some studies revealed that cancer cells isolated from ascites possess stemness properties,^59, 60^ and abundant amount of AREG was also found in ascites of ovarian cancer patients.^61^ Our observation of the overexpression of AREG in ovarian cancer stem-like cells suggesting presence of ovarian cancer stem cells in ascites is consistent with the above mentioned reports. Expression of AREG has been associated with poor prognosis in several cancers, including colon, ovarian, cervical, breast, pancreatic and brain cancers.^15, 62, 63, 64, 65, 66^ Our data indicating that high expression of AREG correlated with advanced stages and shorter survival in ovarian cancer patients is in agreement with previous reports and reinforces that AREG could serve as a prognostic marker for ovarian cancer.

The miR-34 family is composed of three members, including miR-34a, miR-34b and miR-34c.^67, 68^ Although the miR-34 family shares over 80% homology with each other and thus control a similar set of target genes, their differential effects on various cancers and cancer stem cells need further study.^67, 68, 69^ According to our microarray data, only miR-34c-5p in the miR-34 family was downregulated in OVS1 sphere cells compared with their parental cells. The differences of expression in miR-34 family members in different cancers have been previously reported, miR-34a was reported to be overexpressed in renal cell carcinoma tissues, melanoma cell lines, and hepatoma cell lines, yet miR-34c was shown to display low expression in the above tissue samples and cell lines.^70, 71, 72^ We found that miR-34a did not show significant inhibitory effect on AREG. Although miR-34a and miR-34c-5p share the same ‘seed’ region which means base pair between nucleotide 2–7 of the miRNA and complementary sequences in the 3′UTR of the target genes according to the previous reports,^73, 74, 75^ several studies also have indicated that the seed region is not the only factor of target recognition and thus miR-34a and miR-34c-5p exert different regulatory abilities toward their target genes despite their seed regions are identical.^73, 74, 76^ In our study, we confirmed the low expression of miR-34c-5p by qRT–PCR in both SKOV-I6 and OVS1 sphere cells. Low expression of miR-34c has been reported in several cancers including breast, lung, pancreatic, brain, ovarian, laryngeal and prostate cancers.^68, 69, 77, 78, 79, 80^ Some studies also demonstrated downregulation of miR-34c in breast and prostate cancer stem cells and the roles of miR-34c to suppress cancer stemness via targeting p53 and Notch genes but have never linked to AREG.^27, 81, 82^ The role of miR-34c-5p in ovarian cancer stem cells has never been reported before and information on its regulatory role on AREG is also lacking. In this study, we demonstrated that miR-34c-5p directly targeted and downregulated AREG to inhibit ovarian cancer stemness and drug resistance. AREG has been suggested as a good target for treating cancer, inhibition of AREG by siRNA or monoclonal antibodies results in apoptosis of cancer cells or shrinkage of mice xenograft tumors.^15, 61, 83^ Our study showed that transfection of SKOV-I6 cells with miR-34c-5p resulted in significant decrease of tumor volumes in NOD/SCID mice. Our data also demonstrated that miR-34c-5p was able to repress ovarian cancer stemness and drug resistance via targeting AREG-mediated EGFR-ERK pathway. Intercepting EGFR-ERK signaling pathway through blocking EGFR has been widely used in treating colon, breast, lung and head and neck cancers in recent years,^61, 84^ yet the efficacy of blocking EGFR in treating ovarian cancer has not been significant.^85^ Since AREG has been suggested as a promising target in cancer treatment, suppressing AREG to target ovarian cancer stem cells via inhibiting EGFR-ERK signaling pathway could be a reasonable option.^15, 16, 61, 83^ Our results also suggest that targeting AREG by miR-34c-5p might be an alternative strategy to inhibit ovarian cancer stem cells. Further study will focus on the combination efficacy of miR-34c-5p and chemotherapeutic agents, as well as AREG-tailored treatment to target ovarian cancer stem cells.

In the present study, we successfully enriched ovarian cancer stem-like cells and for the first time demonstrated the essential role of AREG in regulating ovarian cancer stemness. We also first revealed that miR-34c-5p directly targeted AREG and downregulated AREG-mediated ovarian cancer stemness and drug resistance. Clinical relevance of AREG with advanced stages and poor outcomes in ovarian cancer patients was reinforced in our study. In conclusion, our data implies that miR-34c-5p could be a promising strategy in the treatment of ovarian cancer via downregulation of the AREG-EGFR-ERK pathway to inhibit ovarian cancer stemness and drug resistance.

## Materials and methods

### Cell lines, cell culture, sphere culture and tumor samples

All patient-related studies were approved by Institutional Review Boards of Taipei Veterans General Hospital and National Health Research Institutes (NHRI). The informed consents were obtained from all patients. The OVS1 cell line was derived from a fresh ovarian tumor obtained from Taipei Veterans General Hospital. The histopathology of the fresh ovarian tumor was described in Supplementary Figure 4. Detailed procedure for derivation of OVS1 cell line was described in Supplementary Figure 7. The highly invasive human ovarian cancer cell line, SKOV-I6, was derive from SKOV-3 cell line reported previously.^86^ The two derived cell lines, SKOV-I6 and OVS1, were both authenticated by STR profiling. Both SKOV-I6 and OVS1 cell were cultured in DMEM (Invitrogen, Carisbad, CA, USA) supplemented with 10% fetal bovine serum (DMEM-10 medium). To obtain spheres in culture, monolayer cells of parental SKOV-I6 and OVS1 cells were cultured in the stem cell selective condition by plating cells in Corning Costar ultra-low attachment 6-well plates (Sigma-Aldrich Inc., St Louis, MO, USA) at a density of 1 × 10^5^ cells per well with 3 ml of serum-free PSGro hESC/iPSC growth medium (System Biosciences, Palo Alto,CA, USA). Propagation of spheres was processed by gentle centrifugation, dissociation with trypsin-EDTA and repeated pipetting to obtain single-cell suspensions, and then plating the cells in the above stem cell selective condition every 5–8 days. The total numbers of spheres were counted under microscope after 10 days of culturing, any sphere consisting of at least 5 cells was calculated according to the published report.^46^ Spheres cultured for 10–14 days were used for all subsequent experiments. Details of tumor samples for IHC stain were described in Figure 7.

### Total RNA isolation and qRT–PCR

Detailed procedures of total RNA isolation and qRT–PCR were described elsewhere.^87^ The expression levels of mRNA were normalized to that of actin or RNU6B. The primer details are described in Supplementary Table 3.

### Western blotting analysis

Detailed procedure was described elsewhere.^87^ All antibodies used are listed in Supplementary Table 4.

### Immunofluorescence staining and confocal microscope image analysis

Immunofluorescence staining was carried out according to the manufacture’s protocol using antibodies against CD44 (BD Biosciences, San Jose, CA, USA), CD133 (Miltenyi Biotec, Auburn, CA, USA), and cytokeratin 7 (CK7) (Abcam, Cambridge, MA, USA) in 1:400 dilution, the Alexa Fluor 488 and Alexa Fluor 594 -conjugated goat anti-mouse IgG antibodies (Invitrogen) were used as the secondary antibodies in 1:200 dilution. Immunofluorescence staining for spheres followed the protocol of a published report.^88^ Samples were inspected and photographed using the Leica SP5 ll scanning confocal microscope (Leica, Bannockburn, IL, USA).

### Cell proliferation assay

The cell proliferation assay was evaluated using MTT assay (Promega, Madison, WI, USA) according to the manufacture’s protocol. Briefly, cells were plated at a density around 5000 cells per well in 96-well plates and were incubated for 24 h. Cells were then treated with different concentrations of docetaxel (TTY Biopharm, Taipei, Taiwan) and carboplatin (Pharmachemie BV, Haarlem, Holland), respectively, and were incubated for 48 h at 37 °C. The quantity of formazan product, which is directly proportional to the number of viable cells, was measured at a wave length of 490 nm with 96-well plate reader. The drug concentration required to suppress proliferation by 50% is defined as IC_50_. All data were calculated from three independent experiments performed in triplicate samples.

### Transfection assay

SKOV-I6 and OVS1 parental cells were cultured until 80% confluence and then were divided into four groups to be transfected with plasmids containing negative control vectors, miR-34c-5p alone, AREG alone, or miR-34c-5p and AREG by using Lipofectamine 2000 (Invitrogen) or RNAi-MAX (Invitrogen). The miR-34c-5p was purchased from Invitrogen. AREG coding sequence was cloned in pcDNA3.1. Plasmids containing the control sequences for miRNA (MDbio, Inc. Taipei, Taiwan) and pcDNA3.1 were used as negative control vectors. Cells were transfected for 48 h and then were collected for appropriate experiments.

### Experimental animals and tumorigenicity test

OVS1 parental cells and spheres dispersed into single-cell suspension were collected and, respectively, divided into four different cell number groups (100, 500, 10^3^ and 10^4^ cells) for injection. Twenty 6- to 8-week-old female NOD/SCID mice (BioLasco, Taipei, Taiwan) were randomly divided into four groups (five mice per group) and were then implanted with the above groups of cells into the right fourth mammary fat pads because fat pad implantation yielded the highest tumor taken rate.^89^ The sample size of each group was to ensure the chance of statistic significance and to minimize the sacrifice of the living animals for humanitarian reasons. Tumor volumes were measured once a week. Tumor generation was evaluated until 9 months after implantation. In addition, parental SKOV-I6 cells (1 × 10^6^ cells) were divided into four groups according to different transfection status described in transfection assay and were implanted randomly into five NOD/SCID mice (BioLasco) for each group as described above. Tumor volumes were measured twice a week. All of the procedures were carried out under approved Institutional Animal Care and Use Committee protocols of NHRI.

### Microarray analysis

Total RNAs were extracted from parental OVS1 cells and their sphere cells and then were analyzed with Affymetrix GeneChip system (Affymetrix, Santa Clara, CA, USA) for gene expression, and analyzed with Affymetrix miRNA 2.0 Arrays (Affymetrix) for miRNA expression. All of the microarray analyses were performed at the Microarry Core Laboratory in NHRI.

### Plasmids construction and 3′UTR luciferase reporter assays

The wild-type AREG-3′UTR was cloned into the pGL3-control plasmid (Promega). The mutant AREG-3′UTR was generated by site directed mutagenesis. The Luc-3′UTR-wt or Luc-3′UTR-mt reporter plasmids were prepared by inserting the AREG-3′UTR-wt carrying a putative miR-34c-5p binding site or its mutant sequence, respectively, into the pGL3-control plasmid. Primer sequences were described in Supplementary Table 3. Luc-AREG-3′UTR-wt or Luc-AREG-3′UTR-mt was cotransfected with the miR-34c-5p plasmid into parental SKOV-I6 and OVS1 cells. The cells were harvested 48 h after transfection. Luciferase activity was measured according to the manufacture’s protocol (Promega). *Renilla* luciferase was cotransfected as a control for normalization.

### IHC

IHC staining was performed according to the published procedure using rabbit anti-AREG antibody (Sigma-Aldrich) at the Pathology Core Laboratory in NHRI.^90^ The IHC score of AREG for each specimen was graded as follows: negative (score 0), weakly positive (score 1), moderately positive (score 2), and strongly positive (score 3).^91, 92^

### Public data sets

As for data set analysis of ovarian cancer patients, we searched the public cancer microarray data set (http://www.oncomine.org) to validate mRNA expression of the AREG gene and its correlation with clinical parameters from The Cancer Genome Atlas – ovarian serous cystadenocarcinoma gene expression data.^93^

### Flow cytometry

The procedures followed the published report.^94^ The cells were stained with anti-human epithelial cell adhesion molecule FITC antibody (Stem Cell Technologies) or with anti-CK7 antibody (Abcam) and were collected and dispersed for flow cytometry using BD FACSAria flow cytometer (BD Biosciences).

### Statistical analysis

Student’s *t*-test was used for comparison of differences between experimental groups. Multiple *t*-test and paired t-test were used for comparison of drug resistance in dose-dependent growth inhibition and for comparison of growth of xenograft tumors between experimental groups. Kaplan–Meier method was used for analyzing survival data. Statistical significance was accepted with *P*<0.05.

## Figures and Tables

**Table 1:** *In vivo* tumorigenicity of parental OVS1 cells and their sphere cells

	*100 cells*	*500 cells*	*10*^*3*^ *cells*	*10*^*4*^ *cells*
OVS1 parental cells	0/5	0/5	0/5	0/5
OVS1 sphere cells	1/5	1/5	1/5	4/5
Tumor latency	135 days	105 days	38 days	23, 30, 30 and 44 days

Abbreviation: OVS1, ovarian tumor-derived cell line.

Note: Tumor generation was evaluated until 9 months after implantation.

## Supplementary Material

Supplementary Figure Lgends
