# Supplementary material for: miRNA-34c-5p inhibits amphiregulin-induced ovarian cancer stemness and drug resistance via downregulation of the AREG-EGFR-ERK pathway
Source: Oncogenesis. 2017 May 1;6(5):e326–. doi: 10.1038/oncsis.2017.25 (PMC5525454; doi:10.1038/oncsis.2017.25)
Supplement: Supplementary Figure Lgends [file oncsis201725x1.docx]

**Supplementary Figure Legend**

**Figure S1.** Expression of ovarian cancer stem cell markers in SKOV-I6 cells. The mRNA expression levels of ovarian cancer stem cell markers in parental SKOV-I6 cells and their sphere cells were analyzed by qRT-PCR with actin as an internal control. Histograms represent means ± SD from three independent experiments (*, *p* < 0.05, **, *p* < 0.01).

**Figure S2.** Comparison of chemoresistance between parental SKOV-I6 and OVS1 cells and their sphere cells. The MTT assay was carried out for parental SKOV-I6 and OVS1 cells and their sphere cells. Left, percentage of viable cells in parental SKOV-I6 cells and their sphere cells treated for 48 hours at IC_50_ level of docetaxel (8 nM) and carboplatin (100 μM). Right, percentage of viable cells in parental OVS1 cells and their sphere cells treated for 48 hours at IC_50_ level of docetaxel (2.5 nM) and carboplatin (150 μM). Histograms represent means ± SD from three independent experiments (*, *p* < 0.05, **, *p* < 0.01).

**Figure S3.** Comparison of chemoresistance between parental SKOV-I6 cells and their sphere cells. Dose-dependent growth inhibition of parental SKOV-I6 cells and their sphere cells upon continuous exposure to the indicated concentrations of docetaxel or carboplatin for 48 hours was measured by MTT assay. Each dosage point represents the mean ± SE from three independent experiments (*, *p* < 0.05).

**Figure S4.** The histopathology of the clinical fresh ovarian tumor which OVS1 cell line was derived from and of a xenograft tumor derived from OVS1 sphere cells. (a) The histopathology showed ovarian metastatic adenocarcinoma of omentum. The neoplastic cells in glandular and individual single cell patterns infiltrating in the omentum tissue. (H&E staining, 100X ). (b) Prominent nuclear atypia was noted in neoplastic cells. (H&E staining, 200X). (c) Apparent mitosis and prominent nucleoli seen in higher magnification. (H&E staining, 400X). (d) These neoplastic cells were strongly positive for cytokeratin (CK staining, 100X). (e) The magnified filed of black frame area of Figure S4b (H&E staining, 400X) (f) The histopathology of a xenograft tumor derived from OVS1 sphere cells (from the group of 500 cells in Figure 1g) was categorized as adenocarcinoma resembling the tumor phenotype (Figure S4e) of the fresh ovarian tumor which OVS1 cell line was derived from (H&E staining, 400X).

**Figure S5.** The expression of miR-34a and its effect on AREG in both SKOV-I6 and OVS1 cell lines. (a) The mRNA expression levels of miR-34a in parental SKOV-I6 and OVS1 cells as well as their sphere cells were analyzed by qRT-PCR. RNU6B was used as an internal control. Histograms represent means ± SD from three independent experiments (NS = no significant differences). (b) MiR-34a did not inhibit AREG mRNA expression levels in SKOV-I6 and OVS1 cells. The mRNA expression levels of AREG in both cell lines were measured by qRT-PCR from cells transfected with the indicated plasmids. Histograms represent means ± SD from three independent experiments (NS = no significant differences).

**Figure S6.** The effects of AREG and miR-34c-5p on drug resistance of SKOV-I6 and OVS1 cells. The MTT assay was carried out for parental SKOV-I6 and OVS1 cells transfected with the indicated plasmids. Left, percentage of viable cells in SKOV-I6 cells treated for 48 hours at IC_50_ level of docetaxel (8 nM) and carboplatin (100 μM). Right, percentage of viable cells in OVS1 cells treated for 48 hours at IC_50_ level of docetaxel (2.5 nM) and carboplatin (100 μM). Histograms represent means ± SD from three independent experiments (*, *p* < 0.05, **, *p* < 0.01).

**Figure S7.** The new cell line OVS1 has been derived from the clinical ovarian

adenocarcinoma specimen. (a) Left, The fresh ovarian tumor was obtained from an

ovarian cancer patient who was diagnosed as ovarian serous adenocarcinoma with

peritoneal carcinomatosis at Taipei Veterans General Hospital. The tumor was

digested to yield single cell suspension followed the procedure described in the

published report.^1^ The dispersed cells were then cultured in monolayer in DMEM

(Invitrogen) supplemented with 10% fetal bovine serum (DMEM-10 medium) for 4

passages before sorting of ESA^+^ (epithelial specific antigen) cells. Right, the ESA^+^

cells isolated by flow cytometry were then cultured in monolayer in DMEM-10

medium continuously for more than 100 generations and became an immortalized cell

line, which we named OVS1. (b) The ovarian fresh tumor derived cells of Figure S6a

(left column) were subsequently sorted for the ESA^+^ cells using anti-human epithelial

cell adhesion molecule FITC antibody by flow cytometry for isolation of epithelial

cells. (c) The ESA^+^ cells were stained with cytokeratin 7(CK7) shown by both flow

cytometry (top) and confocal immunofluorescence (bottom) to confirm the primary

ovarian cancer status.^2^ The nuclei were stained with DAPI.

**References for Supplementary Figure legend**

1. Kreso A, O'Brien CA. Colon cancer stem cells. *Current protocols in stem cell*

*biology* 2008; **Chapter 3**: Unit 3 1

2. Kriplani D, Patel MM. Immunohistochemistry: A diagnostic aid in differentiating

primary epithelial ovarian tumors and tumors metastatic to the ovary. *South Asian*

*J Cancer* 2013; **2**: 254-258.
