# Supplementary figures and images for: miRNA-34c-5p inhibits amphiregulin-induced ovarian cancer stemness and drug resistance via downregulation of the AREG-EGFR-ERK pathway
Source: Oncogenesis. 2017 May 1;6(5):e326–. doi: 10.1038/oncsis.2017.25 (PMC5525454; doi:10.1038/oncsis.2017.25)

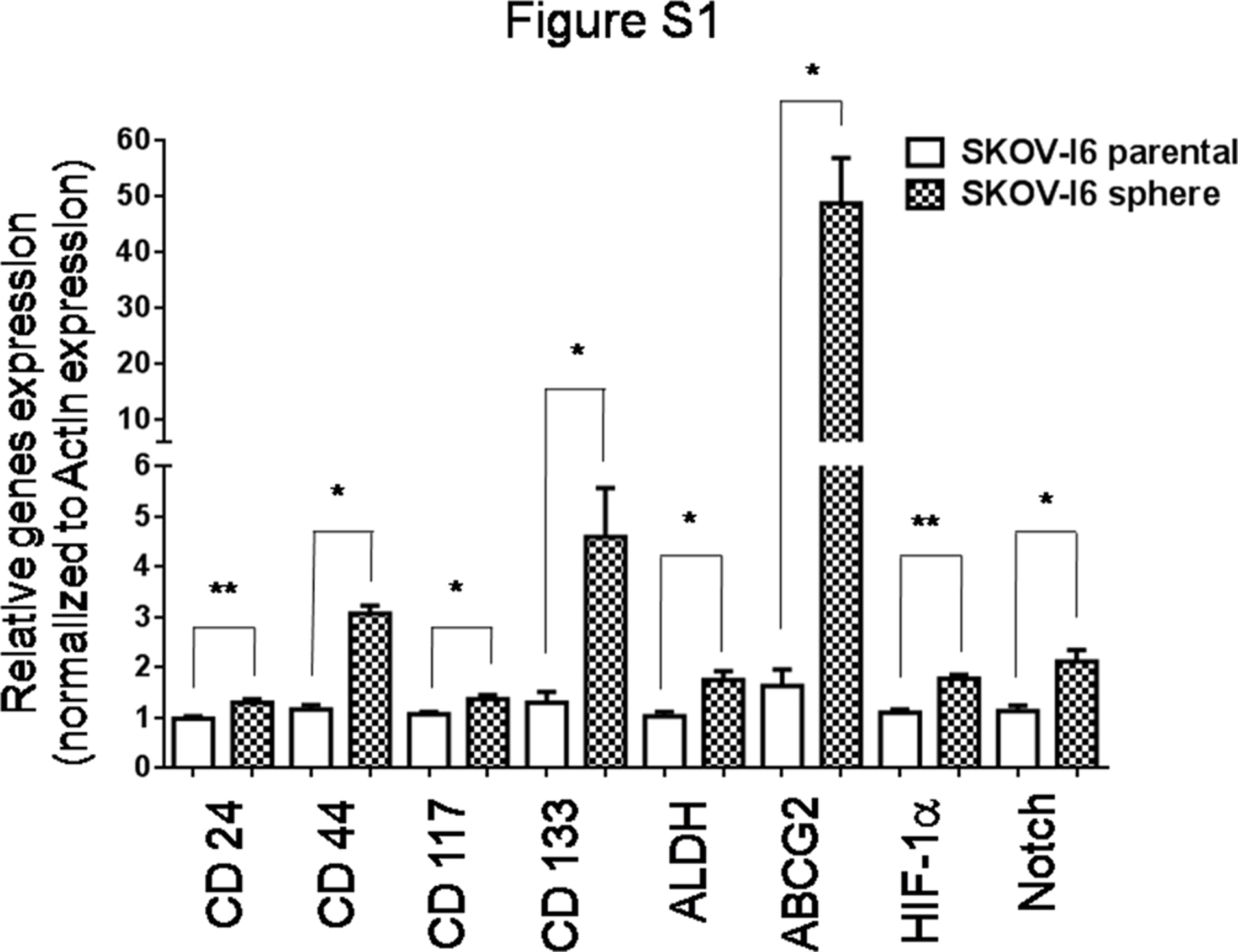

Supplement: Supplementary Figure S1 [file oncsis201725x2.tif]

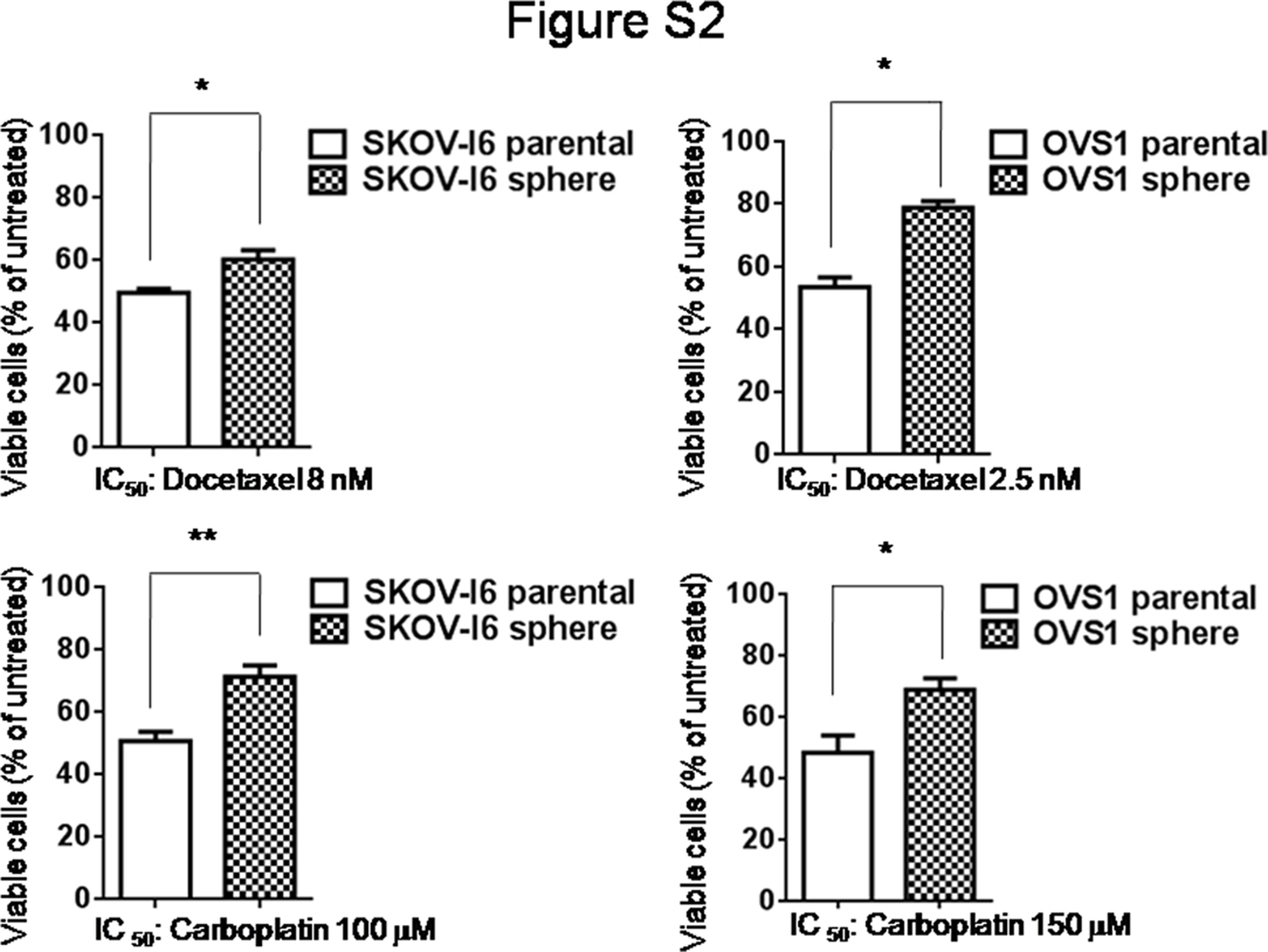

Supplement: Supplementary Figure S2 [file oncsis201725x3.tif]

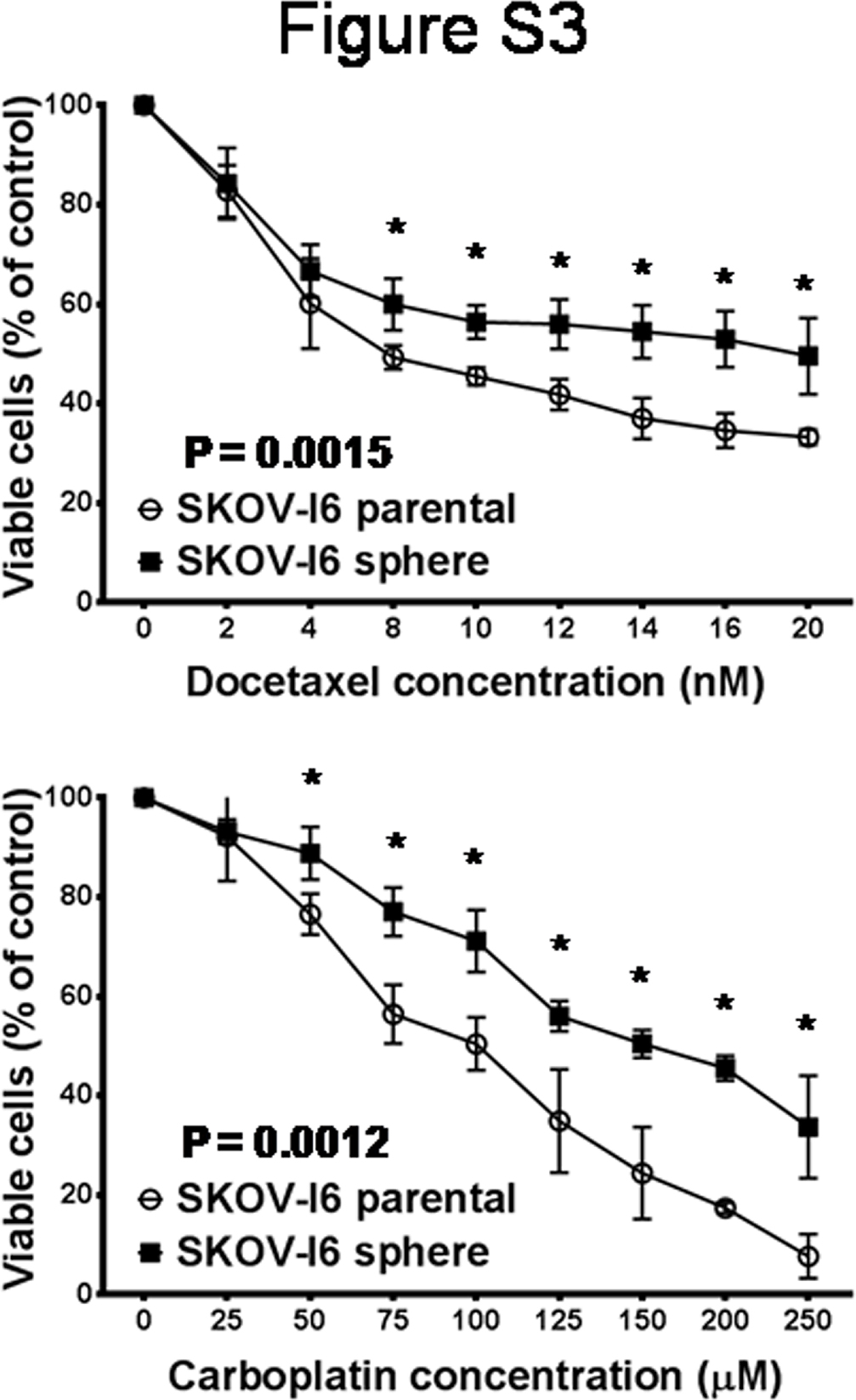

Supplement: Supplementary Figure S3 [file oncsis201725x4.tif]

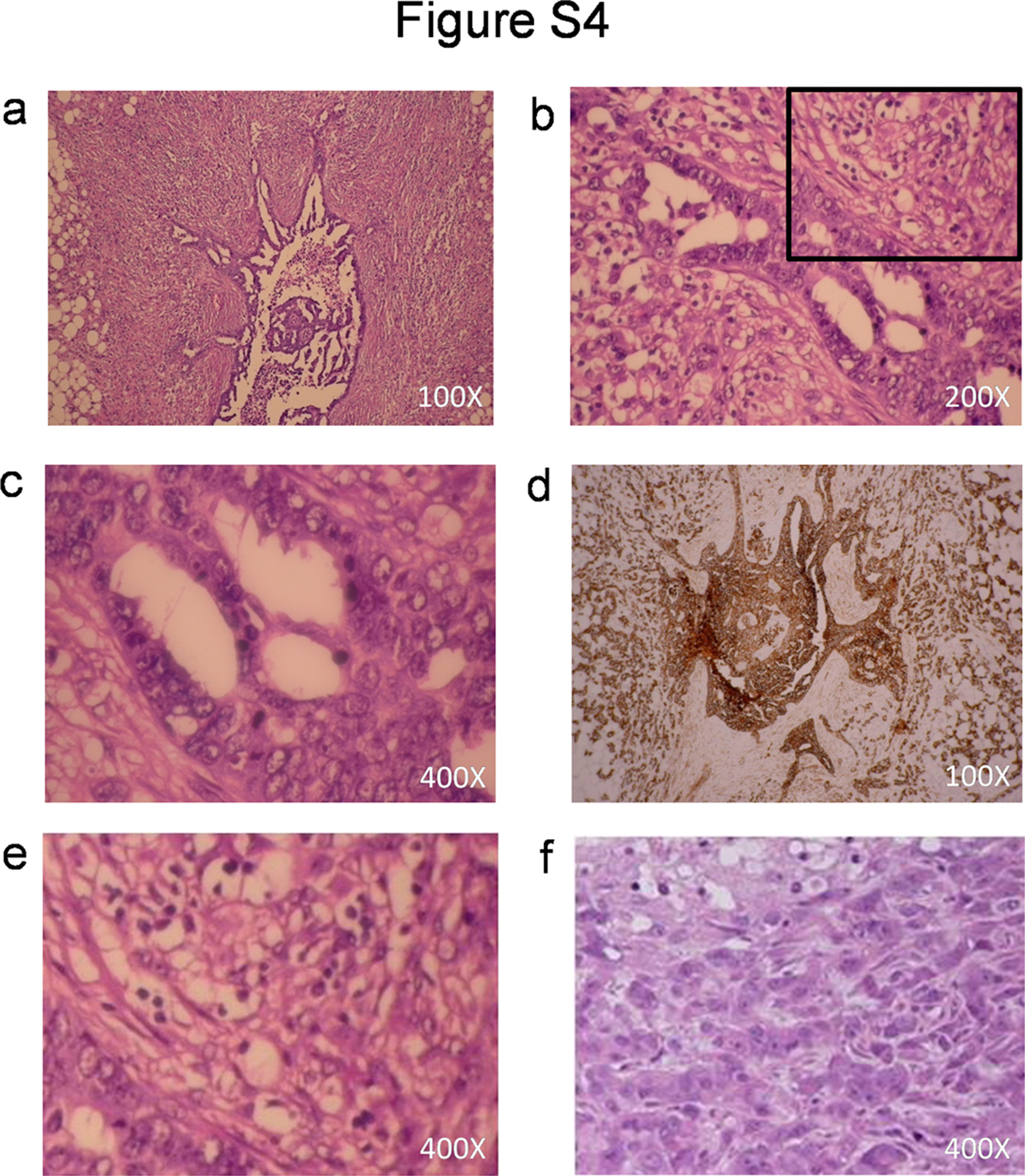

Supplement: Supplementary Figure S4 [file oncsis201725x5.tif]

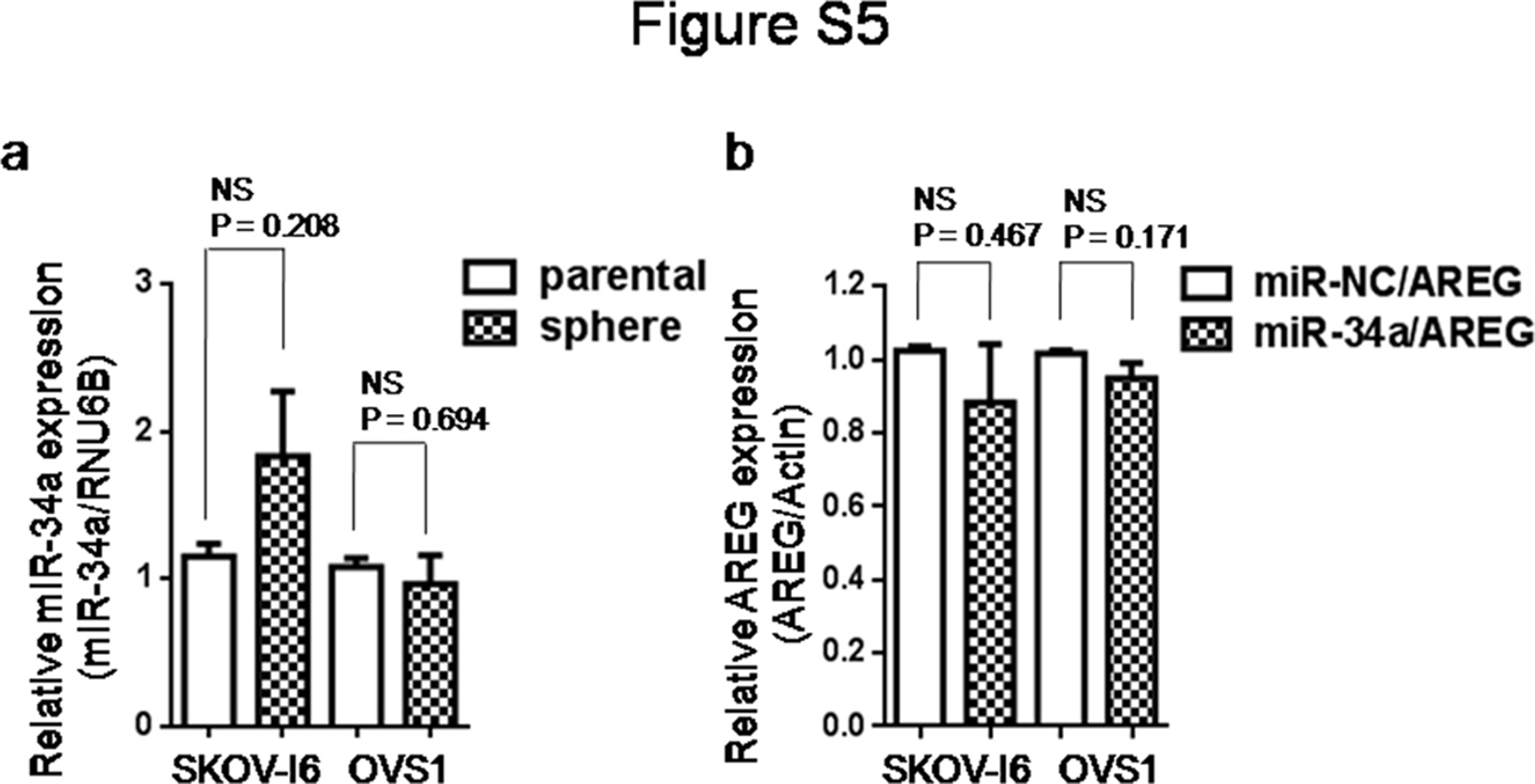

Supplement: Supplementary Figure S5 [file oncsis201725x6.tif]

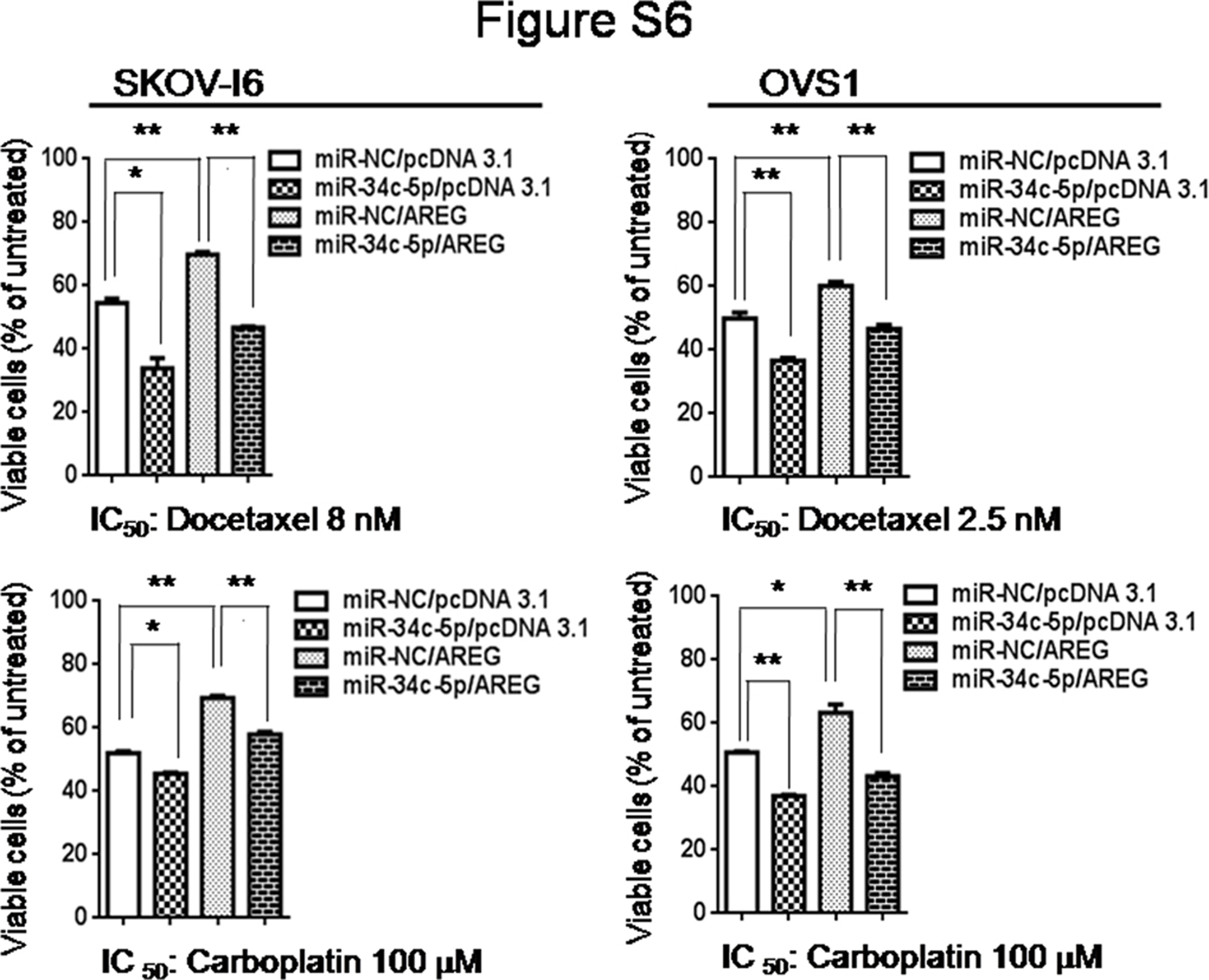

Supplement: Supplementary Figure S6 [file oncsis201725x7.tif]

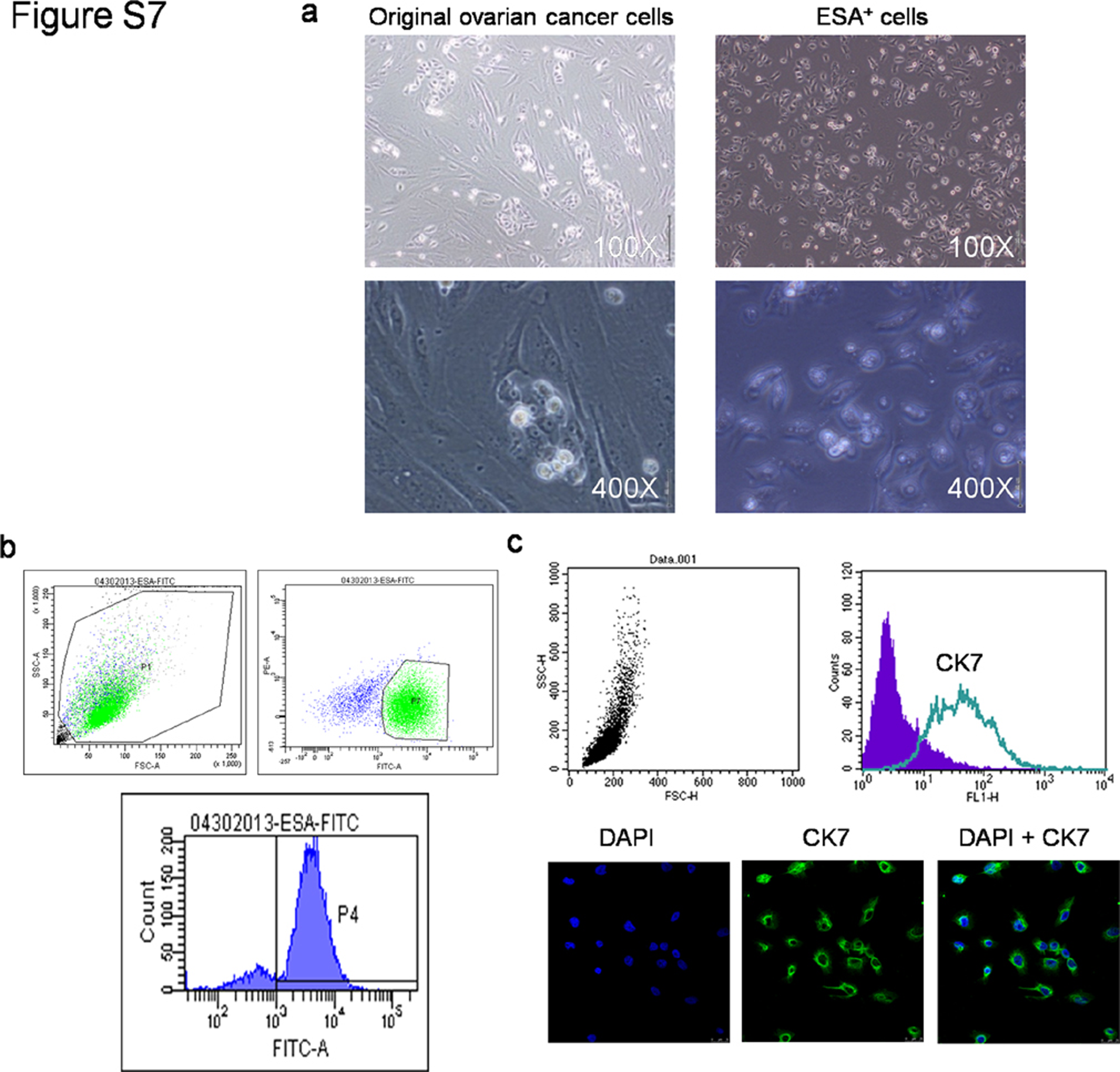

Supplement: Supplementary Figure S7 [file oncsis201725x8.tif]
