# Supplementary material for: miRNA-34c-5p inhibits amphiregulin-induced ovarian cancer stemness and drug resistance via downregulation of the AREG-EGFR-ERK pathway
Source: Oncogenesis. 2017 May 1;6(5):e326–. doi: 10.1038/oncsis.2017.25 (PMC5525454; doi:10.1038/oncsis.2017.25)
Supplement: Supplementary Table S1 [file oncsis201725x9.docx]

**Supplementary Table S1. The conserved miRNAs of 11 upregulated cancer stem cells related genes of OVS1 spheres predicted by TargetScan 7.0.**

| Cancer stem cell related genes | Conserved miRNAs | Predicted consequential pairing of target region (top) and miRNA (bottom) | Site type |
| --- | --- | --- | --- |
| DPP4 | Position 519-526 of DPP4 3' UTR  hsa-miR-6504-5p | 5' ...GAGAAGAGCUGUUCACAGCCAGA...                     \|\|\|\|\|\|\|\|\|  3'       GACGUAAUGUCGUGUCGGUCU | 8mer |
|  | Position 519-526 of DPP4 3' UTR  hsa-miR-3064-5p | 5'  ...GAGAAGAGCUGUUCACAGCCAGA...                     \|\| \| \|\|\| \|  3'        AACGUGUGGUGUUGUCGGUCU | 8mer |
| AREG | Position 35-42 of AREG 3' UTR  hsa-miR-34c-5p | 5'  ...AUAUCACAUUGGAGUCACUGCCA...                     \|\|\| \| \|\| \|  3'     CGUUAGUCGAUUGAUGUGACGGA | 8mer |
|  | Position 35-42 of AREG 3' UTR  hsa-miR-34a-5p | 5' ...AUAUCACAUUGGAGUCACUGCCA...                    \|\| \|\|\| \| \|  3'     UGUUGGUCGAUUCUGUGACGGU | 8mer |
|  | Position 35-42 of AREG 3' UTR  hsa-miR-449b-5p | 5'    ...AUAUCACAUUGGAGUCACUGCCA...                       \|\| \|\| \| \|\|  3'        CGGUCGAUUGUUAUGUGACGGA | 8mer |
|  | Position 35-42 of AREG 3' UTR  hsa-miR-449a | 5'    ...AUAUCACAUUGGAGUCACUGCCA...                           \|\|\| \|\| \|\|  3'         UGGUCGAUUGUUAUGUGACGGU | 8mer |
| EDIL3 | Position 421-428 of EDIL3 3' UTR  hsa-miR-137 | 5'   ...GAAUACUGCAAUGUUAGCAAUAA...                        \| \|\| \|\|\| \|  3'      GAUGCGCAUAAGAAUUCGUUAUU | 8mer |
| ITGA2 | Position 270-276 of ITGA2 3' UTR  hsa-miR-30b-5p | 5'  ...ACUGGCUGGCCCAGAGUUUACAU...                      \|\|\|\|\|\|   3'    UCGACUCACAUCCUACAAAUGU | 7mer-A1 |
|  | Position 270-276 of ITGA2 3' UTR  hsa-miR-30c-5p | 5'   ...ACUGGCUGGCCCAGAGUUUACAU...                      \|\|\|\|\|\|   3'     CGACUCUCACAUCCUACAAAUGU | 7mer-A1 |
|  | Position 270-276 of ITGA2 3' UTR  hsa-miR-30d-5p | 5'   ...ACUGGCUGGCCCAGAGUUUACAU...                        \|\|\|\|\|\|   3'       GAAGGUCAGCCCCUACAAAUGU | 7mer-A1 |
|  | Position 270-276 of ITGA2 3' UTR  hsa-miR-30e-5p | 5'    ...ACUGGCUGGCCCAGAGUUUACAU...                          \|\|\|\|\|\|   3'       GAAGGUCAGUUCCUACAAAUGU | 7mer-A1 |
|  | Position 270-276 of ITGA2 3' UTR  hsa-miR-30a-5p | 5'  ...ACUGGCUGGCCCAGAGUUUACAU...                        \|\|\|\|\|\|   3'     GAAGGUCAGCUCCUACAAAUGU | 7mer-A1 |
| MAOA | Position 2274-2281 of MAO A 3' UTR  hsa-miR-495-3p | 5'   ...GUAUGGUACUGUUUUGUUUGUUA...                         \| \| \| \| \| \|\|  3'     UUCUUCACGUGGUACAAACAAA | 8mer |
|  | Position 2274-2281 of MAO A 3' UTR  hsa-miR-5688 | 5'  ...GUAUGGUACUGUUUUGUUUGUUA...      \|\|\|        \|\|\| \|\|\|\|  3'   CGACAAAAUGUCCA-----CAAACAAU | 8mer |
| EGR1 | Position 411-417 of EGR1 3' UTR  hsa-miR-183-5p.1 | 5'     ...CCUUGUACAGUGUCUGUGCCAUG...                           \|\| \| \|\|\|\|  3'          UCACUUAAGAUGGUCACGGUAU | 7mer-m8 |
| TMPRSS4 | Position 114-121 of TMPRSS4 3' UTR  hsa-miR-485-5p | 5' ...ACACCCCUCUGCCCACAGCCUCA...                      \|\| \| \|\| \|\|  3'     CUUAAGUAGUGCCGGUCGGAGA | 8mer |
|  | Position 114-121 of TMPRSS4 3' UTR  hsa-miR-6884-5p | 5'   ...ACACCCCUCUGCCCACAGCCUCA...                         \|\|\| \|\| \|\|  3'       GUUGUAGUGGAAGAGUCGGAGA | 8mer |
| CD109 | Position 4098-4104 of CD109 3' UTR  hsa-miR-203a-3p.1 | 5' ...AUAUGUUGUCAUUUUCAUUUCAG...               \|\|\|      \|\|\|\|\|\|   3'     AUCACCAGGAUUUGUAAAGU | 7mer-A1 |
| ABCA12 | Position 221-228 of ABCA12 3' UTR  hsa-miR-3681-3p | 5'  ...UUCCUGUAUACUCAACACUGUGA...                       \|\|\| \|\| \|\|  3'       UCAUCACCUACUUCGUGACACA | 8mer |
|  | Position 221-228 of ABCA12 3' UTR  hsa-miR-128-3p | 5'  ...UUCCUGUAUACUCAACACUGUGA...                        \| \|\| \| \| \| \|  3'      UUCUCUGGCCAAGUGACACU | 8mer |
|  | Position 221-228 of ABCA12 3' UTR  hsa-miR-216a-3p | 5'    ...UUCCUGUAUACUCAACACUGUGA...                         \|\|\| \| \| \|\|  3'        UAUUAGGGUCUCUGGUGACACU | 8mer |
| CD44 | Position 131-137 of CD44 3' UTR  hsa-miR-199a-3p | 5' ...AACAGAUGCAAUGUG--------CUACUGAU...            \|\| \|\| \| \|\|      \|\|\| \| \|\|   3'          AUUGGUUACACGUCUGAUGACA | 7mer-A1 |
|  | Position 131-137 of CD44 3' UTR  hsa-miR-199b-3p | 5' ...AACAGAUGCAAUGUG--------CUACUGAU...                \| \|\| \| \|\| \|     \| \|\| \| \| \|   3'         AUUGGUUACACGUCUGAUGACA | 7mer-A1 |
|  | Position 131-137 of CD44 3' UTR  hsa-miR-3129-5p | 5'  ...AACAGAUGCAAUGUGCUACUGAU...                        \|\|\| \|\| \|   3'     UUUGGUUAGAGAUGUGAUGACG | 7mer-A1 |
| CFB | No conserved miRs were found |  |  |
