# Supplementary material for: miRNA-34c-5p inhibits amphiregulin-induced ovarian cancer stemness and drug resistance via downregulation of the AREG-EGFR-ERK pathway
Source: Oncogenesis. 2017 May 1;6(5):e326–. doi: 10.1038/oncsis.2017.25 (PMC5525454; doi:10.1038/oncsis.2017.25)
Supplement: Supplementary Table S2 [file oncsis201725x10.docx]

**Supplementary Table S2. Fold change of the predicted conserved miRNAs in OVS1 sphere cells compared with their parental cells**

|  | Predicted conserved miRNAs | Fold change of miRNAs | Target genes |
| --- | --- | --- | --- |
| Upregulated miRNAs | hsa-miR-137 | 2.65 | EDIL3 |
|  | hsa-miR-199a-3p | 3.07 | CD44 |
|  | hsa-miR-34a | 2.51 | AREG |
| Downregulated miRNAs | hsa-miR-34c-5p | -2.26 | AREG |

Note: The fold change of expression level of miR-34c-5p in OVS1 sphere cells is -2.26 compared with their parental cells, namely the level of miR-34c-5p in OVS1 sphere cells is about 44% of that of their parental cells.
