# Supplementary material for: miRNA-34c-5p inhibits amphiregulin-induced ovarian cancer stemness and drug resistance via downregulation of the AREG-EGFR-ERK pathway
Source: Oncogenesis. 2017 May 1;6(5):e326–. doi: 10.1038/oncsis.2017.25 (PMC5525454; doi:10.1038/oncsis.2017.25)
Supplement: Supplementary Table S3 [file oncsis201725x11.docx]

**Supplementary Table S3. Primers used in this study**

| **Luc- 3’UTR sequence for miR-34c-5p binding site** | | | | | |
| --- | --- | --- | --- | --- | --- |
| Putative miR-34c-5p binding site | | | | 5'-AUAUCACAUUGGAGUCACUGCCA-3' | |
| Mutant miR-34c-5p binding site | | | | 5' -AUAUCACAUUGGAGUUCUGAUUA-3' | |
| **Oligonucleotide sequences used for qRT-PCR** | | | | | |
| **Gene** | **Forward primer (5’-3’)** | | | | **Reverse primer (5’-3’)** |
| CD24 | CTGCTGGCACTGCTCCTAC | | | | ACCACGAAGAGACTGGCTGT |
| CD44 | AGCAACCAAGAGGCAAGAAA | | | | GTGTGGTTGAAATGGTGCTG |
| CD117 | CGTCCAGACAGGCTCTTCTC | | | | GTTGGTGCACGTGTATTTGC |
| CD133 | TTGTGGCAAATCACCAGGTA | | | | TCAGATCTGTGAACGCCTTG |
| ALDH | ACAATGGCAAGCCCTATGTC | | | | ACAGGTTCATGGCGTGTGTA |
| ABCG2 | CACAGGTGGAGGCAAATCTT | | | | CCGAAGAGCTGCTGAGAACT |
| HIF1-α | CCACCTATGACCTGCTTGGT | | | | TATCCAGGCTGTGTCGACTG |
| Notch | GCCAGAGTGGACAGGTCAGT | | | | AATGTTCTCGCTGCAGTCCT |
| Vimentin | CAGATGCGTGAAATGGAAGA | | | | TCCAGCAGCTTCCTGTAGGT |
| Slug | GAGCATTTGCAGACAGGTCA | | | | TCCTCATGTTTGTGCAGGAG |
| Snail | GCGAGCTGCAGGACTCTAAT | | | | CCCACTGTCCTCATCTGACA |
| Twist | AGTCCGCAGTCTTACGAGGA | | | | CATCTTGGAGTCCAGCTCGT |
| E-cadherin | GCTGGAGATTAATCCGGACA | | | | ACCTGAGGCTTTGGATTCCT |
| AREG | TGGATTGGACCTCAATGACA | | | | AGCCAGGTATTTGTGGTTCG |
| miR-34a | GGTGGCAGTGTCTTAGCT | | | | GTGCAGGGTCCGAGGT |
| miR-34c-5p | CGGAGGCAGTGTAGTTAGCT | | | | GTGCAGGGTCCGAGGT |
| RNU6B | TTCCTCCGCAAGGATGACACGC | | | | GTGCAGGGTCCGAGGT |
| Actin | CGGCATCGTCACCAACTG | | | | TCTCAAACATGATCTGGGTCATCT |
| Tubulin | CTCTCCGTCCATCAGTTGGT | | | | TGGTGTGGTCAGCTTCAGAG |
| **Gene RT-primer sequences** | | | | | |
| miR-34a | GTTGGCTCTGGTGCAGGGTCCGAGGTATTCGCACCAGAGCCAACACAACC | | | | |
| miR-34c-5p | GTTGGCTCTGGTGCAGGGTCCGAGGTATTCGCACCAGAGCCAACGCAATC | | | | |
| RNU6B | GTTGGCTCTGGTGCAGGGTCCGAGGTATTCGCACCAGAGCCAACAAAAATAT | | | | |
| **Primer used for plasmid construction** | | | | | |
| **Plasmid**  **Primer sequences (5’-3’)** | | | | | |
| AREG-pcDNA3.1 | | Forward primer | CTGCGAAGGACCAATGAGAG | | |
|  | | Reverse primer | GGCAGTGACTCCAATGTGAT | | |
| Luc-AREG 3’-UTR-wt | | Forward primer | CAGTCCAGCTTAGAAGACAAT | | |
|  |  | Reverse primer | CATTGTCTTATGATCCACTGG | | |
| Luc-AREG 3’-UTR-mt | | Forward primer | ATATCACATTGGAGTTCTGATTAAGTCATAGCCATAA | | |
|  |  | Reverse primer | TTATGGCTATGACTTAATCAGAACTCCAATGTGATAT | | |
