# Supplementary material for: miRNA-34c-5p inhibits amphiregulin-induced ovarian cancer stemness and drug resistance via downregulation of the AREG-EGFR-ERK pathway
Source: Oncogenesis. 2017 May 1;6(5):e326–. doi: 10.1038/oncsis.2017.25 (PMC5525454; doi:10.1038/oncsis.2017.25)
Supplement: Supplementary Table S4 [file oncsis201725x12.docx]

**Supplementary Table S4. All antibodies used in Western blotting**

| **Antibodies Vendor Catalog number** | | |
| --- | --- | --- |
| anti-Vimentin antibody | Abcam,  Cambridge, MA, USA | ab8978 |
| anti-Snail antibody | Abcam,  Cambridge, MA, USA | ab53519 |
| anti-Twist antibody | Santa Cruz Biotechnology, Santa Cruz, CA, USA | sc-81417 |
| anti-E-cadherin antibody | Abcam,  Cambridge, MA, USA | ab15148 |
| anti-AREG antibody | R&D Systems, Inc., Minneapolis, MN, USA | BAF262 |
| anti-EGFR antibody | Cell Signaling Technology, Danvers, MA, USA | 4267S |
| anti-p-EGFR antibody | Cell Signaling Technology, Danvers, MA, USA | 2234S |
| anti-Raf antibody | Cell Signaling Technology, Danvers, MA, USA | 9422S |
| anti-p-Raf antibody | Cell Signaling Technology, Danvers, MA, USA | 9421S |
| anti-ERK antibody | Cell Signaling Technology, Danvers, MA, USA | 4695S |
| anti-p-ERK antibody | Cell Signaling Technology, Danvers, MA, USA | 9101S |
| anti-p44/42 MEK antibody | EMD Millipore, Temecula, CA, USA | NG1915288 |
| anti-p- p44/42 MEK antibody | Calbiochem, Novabiochem, and Novagen, Temecula, CA, USA | 444955 |
| anti-β-actin antibody | Santa Cruz Biotechnology, Santa Cruz, CA, USA | sc-47778 |
| anti-tubulin antibody | Santa Cruz Biotechnology, Santa Cruz, CA, USA | sc-398103 |
